# Abdominal obesity is strongly associated with Cardiovascular Disease and its Risk Factors in Elderly and very Elderly Community-dwelling Chinese

**DOI:** 10.1038/srep21521

**Published:** 2016-02-17

**Authors:** Huimin Fan, Xiaolin Li, Liang Zheng, Xiaoli Chen, Qin lan, Hong Wu, Xugang Ding, Dingguang Qian, Yixin Shen, Zuoren Yu, Lieying Fan, Ming Chen, Brian Tomlinson, Paul Chan, Yuzhen Zhang, Zhongmin Liu

**Affiliations:** 1 Research Center for Translational Medicine, Key Laboratory of Arrhythmias, Ministry of Education, Shanghai East Hospital, Tongji University School of Medicine, Shanghai, 200120, China; 2 Gaohang Community Medical Center, 180 Gingao Road, Pudong New area, Shanghai, 200120, China; 3 Department of Medicine and Therapeutics, The Chinese University of Hong Kong, Hong Kong SAR, China; 4 Division of Cardiology, Department of Internal Medicine, Wan Fang Hospital, Taipei Medical University, Taipei, Taiwan

## Abstract

Obesity is usually considered to predispose to atherosclerotic cardiovascular disease (ASCVD) but milder degrees of obesity or overweight may be protective in some elderly populations. We examined the relationships between general and abdominal obesity indices with ASCVD and its risk factors in elderly (aged ≥65 years) Shanghai community residents Among the 3950 participants, 21.5% had ASCVD, 56.2% had body mass index (BMI) ≥24 kg/m2, 50.1% had high waist circumference (WC) and 77.1% had waist-to-height ratio (WHtR) ≥0.50. WHtR increased with age in both men and women whereas WC increased with age only in women and BMI decreased with age only in men. The optimal WHtR cut-off value to predict the risk of ASCVD determined by receiver operating characteristic analysis was WHtR ≥0.53 with a prevalence of 55.8%. Having abdominal obesity was significantly associated with prevalent ASCVD with WHtR ≥0.53 having a higher value for the odds ratio than high WC, whereas high BMI was not associated. All three indices predicted high glucose, triglycerides and hsCRP levels but only the WHtR ≥0.53 showed a significant association with physical activity. Abdominal obesity indices, but not BMI, predicted prevalent ASCVD and its risk factors in this elderly Chinese population.

Cardiovascular disease (CVD) is the leading cause of death in most countries worldwide. Over the past 3 decades, the prevalence of the CVD risk factors hypertension and raised plasma cholesterol levels have declined in many high-income countries, but the prevalence of overweight and obesity continued to increase in both developed and developing countries^1^,^2^,^3^. Excess adiposity is associated with reduced physical activity and several chronic conditions including type 2 diabetes, hypertension, and dyslipidaemia which lead to a high prevalence of CVD, and an increase in obesity-related morbidity has imposed a heavy burden in many health care systems^3^,^4^,^5^,^6^.

Body mass index (BMI) has served as the classical proxy for general overweight and obesity for many years^1^,^3^,^4^,^5^,^6^,^7^, but emerging clinical and epidemiological evidence indicate that markers of the central distribution of adiposity, such as waist circumference (WC) and especially the waist-to-height ratio (WHtR), show stronger associations with CVD and CVD risk factors or improve the identification of subjects with these problems^8^,^9^,^10^,^11^,^12^. The prospective Coronary Artery Risk Development in Young Adults (CARDIA) study of over 4000 young adults in the United States with a median follow up of 24.8 years^13^, a study of 46,024 adult participants in a nationally representative sample in China surveyed in 2007–2008^14^, an analysis of 244,266 Chinese adults from seven nationwide health examinations^15^, and a study of a mixed ethnic group of 1,891 subjects in Singapore^16^ all concluded that the combination of BMI and abdominal obesity measures were better predictors of CVD and the CVD risk factors than BMI or WC/WHtR alone. However, most studies have focused on young and middle-aged populations and data in the elderly, especially the very elderly aged over 80 years, are relatively sparse.

One cohort study of elderly Spanish men and women at high cardiovascular risk found that abdominal obesity measured by WC or WHtR had higher discriminative ability than BMI or body weight for diabetes mellitus, high fasting plasma glucose, atherogenic dyslipidaemia and metabolic syndrome^11^. However, a recent prospective study in patients with type 2 diabetes found that being overweight or obese was associated with a higher risk for cardiovascular events though being overweight but not being obese was associated with a lower mortality risk^17^. Being overweight and having abdominal obesity was not associated with a higher risk of death and was even associated with decreased all-cause mortality in some studies of elderly populations, including nonagenarians in the Vitality 90 plus, community-dwelling elderly populations^7^,^18^,^19^,^20^. Therefore, it suggests that a paradox exists between overweight/obesity and the true level of risk it poses in terms of cardiovascular events and mortality in elderly Chinese populations. To our knowledge there is no report evaluating the association of overweight/obesity and the distribution of adiposity with ASCVD and risk factors in an elderly urban community population in China, especially including the very elderly aged over 80 years.

China has experienced the world’s fastest economic development over the past three decades, while Shanghai has been one of the fastest developing cities in the world for the last twenty years resulting in a tremendous growth of CVD risk factors and their effects on cardiovascular health^21^. The prevalence of overweight and obesity in China has increased more rapidly than in Western countries^2^, and combined with rapid population aging, older adults will bear two-thirds of the total disease burden in China by 2030^22^. The Shanghai Elderly Cardiovascular Health Study (SHECHS) was conducted to recruit elderly residents aged 65 years and over in Shanghai to provide current and reliable data for evaluating the prevalence of CVDs and their major risk factors to assist in future management to reduce CVD events. This article describes the prevalence of general overweight and obesity and abdominal obesity and explores which obesity indices (BMI, WC or WHtR) are better associated with clinical atherosclerotic CVD (ASCVD), the risk factors including blood pressure, fasting glucose, HbA1c, lipids, uric acid and high-sensitivity C reactive protein (hsCRP) and protecting factors including greater physical activity and higher education level in this elderly community population of Shanghai, China.

## Methods

### Data collection

The study was conducted in accordance with the Declaration of Helsinki and the Institutional Review Board of Tongji Medical School affiliated Shanghai East Hospital approved the study protocol. Written informed consent was obtained from each participant before any sample or data collection.

The SHECHS was a community population-based, longitudinal study of non-institutionalized older individuals. Gaohang community is located in the northeast part of Pudong New Area of Shanghai China, east of Waigaoqiao Free Trade Zone and west of the Huangpu River. There are 29 communities in the town and the populations have similar availability of medical care but differ in the education level of inhabitants. All subjects aged 65 years and older who were permanent residents in the Gaohang community were invited to participate and those consenting to participate were instructed to come to the medical center in the morning after at least 10 hours of overnight fast. Blood samples were obtained on their arrival at the center and sent to the Blood Laboratory of Tongji Medical School affiliated Shanghai East Hospital for processing and measurement within 2 hours. Fasting serum glucose, lipid profile and uric acid were measured enzymatically on the Roche Cobas 8000 C702 Biochemistry system. HbA1c was measured by ion-exchange high-performance liquid chromatography on the ToSoH G8 analyzer and hsCRP was measured by particle enhanced immunonephelometry.

After blood sampling, the participants were interviewed by trained family doctors using a standard questionnaire to obtain information on demographic characteristics, personal and medical history and lifestyle risk factors and this was followed by recording the ECG and the two-dimensional echocardiography was performed and measured by trained specialists of Shanghai East Hospital using a color Doppler ultrasonic system equipped with a 1.0–5.0 MHz transducer (GE Vivid 7; General Motors Corporation, New York, USA). Anthropometric measurements were obtained according to a standardized protocol including blood pressure (BP), body weight, height, and WC^23^.

### Study-outcome definitions

Body mass index (BMI) was defined as weight in kilograms divided by height in meters squared. Participants were classified as being generally overweight (BMI ≥24 and <28 kg/m2) or obese (BMI ≥28 kg/m2) according to the Asian-specific BMI cut-points of the Health Standard of the People’s Republic of China National Health and Family Planning Commission^12^,^24^, while abdominal obesity was defined by WC ≥90 cm in men and ≥85 cm in women^12^ or WHtR ≥0.5 according to previous reports^25^,^26^. We performed receiver operating characteristic (ROC) analysis to further determine the optimal WHtR cutoff point in this elderly Chinese community population. The amount of physical activity was determined by a questionnaire and designated as active when exercise or recreation activity was performed at least 4 days per week and for more than 30 minutes every day, or not active when the activity did not meet these criteria. Clinical ASCVD was defined as having a history of myocardial infarction (MI), coronary or other arterial revascularization, stable or unstable angina, stroke, transient ischaemic attack (TIA) or peripheral artery disease and was confirmed by review of the outpatient medical records of primary care in the community health centers^27^.

### 10-year estimated risk of ischaemic cardiovascular diseases

Estimates of lifetime risk of CVD can provide a comprehensive assessment of the overall burden of the disease in the population currently and in the future and help guide health care services to reduce the disease events. We used an equation validated by the USA-PRC Collaborative Study of Cardiovascular and Cardiopulmonary Epidemiology Research Group and the China Multicenter Collaborative Study of Cardiovascular Epidemiology (China MUCA) Research Group, which used traditional covariates age, systolic BP (SBP), BMI, total cholesterol (TC), diabetes and smoking, to estimate the 10-year risk of ischaemic CVD of the elderly population^28^. The ischaemic CVDs included MI, angina, stroke and TIA as clinical ASCVD. We estimated the optimal value of WHtR in this elderly Chinese community population to identify association with increased risk of ASCVD.

### Statistical Analysis

Descriptive statistics were calculated for all variables and significant differences in continuous variables were determined by ANOVA and Student’s t-test, and categorical percentile values were compared by Chi-squared test (χ^2^-test). The ROC analysis was performed to determine an optimal WHtR value to discriminate between individuals with intermediate and high 10-year estimated risk of ischaemic CVD from low-risk individuals. Then, the value with the highest sum of sensitivity and specificity (Youden index) was identified as the cut-off point. Multivariate logistic regression analyses were used to examine the association of CVD risk factors with odds ratios (ORs) and 95% confidence intervals (CIs) of general overweight and obesity defined by BMI or abdominal obesity by WC or WHtR. All statistical analyses were performed using SPSS17.0 software (SPSS Inc., Chicago, IL, USA) and a two-tailed P value < 0.05 was considered to be statistically significant.

## Results

### Demographic and clinical characteristics of SHECHS participants

A total of 3950 participants including 1745 males and 2205 females aged ≥65 years completed the SHECHS baseline examination and the demographic data are shown in Table 1. The total population size aged ≥65 years in the community is approximately 8,500. The education level of participants was 10.17% of grade 12 or higher and the proportion with education level at this grade was lower in women (4.63%) than in men (17.18%). More men than women were self-reported as being physically active with 28.46% of men regarded as active compared to 21.90% of women (P < 0.001). The prevalence of clinical ASCVD in SHECHS participants was 21.51% overall with no significant difference between genders. However, a significant increase in prevalence of ASCVD was found with increasing age, with 17.56%, 24.46%, 31.17% of men and 16.29%, 25.45%, 24.37% of women having ASCVD in the 65–69, 70–79 and over 80 year age groups, respectively (Table 2, P < 0.001).

### Mean levels of BMI, waist circumference, waist-to height ratio and other metabolic variables

Body height and weight was significantly greater in men than women but there was no significant difference in BMI between genders. With increasing age there was a significant decline in body weight and height in both genders (Table 2, P < 0.001), and a significant decline in BMI only in men (Table 2, P < 0.05). Overall WC was greater in men than women and increased with age in both genders, but significantly only in women, so there was no difference of WC between men and women in those over 80 years of age (Table 2). WHtR increased significantly with age in both genders and was greater in women than men in all age groups (Table 2, p < 0.001).

In both genders, with increasing age there were significant increases of ASCVD, SBP, high-density lipoprotein (HDL)-cholesterol, uric acid and hsCRP, but significant decreases of triglyceride (TG) and no change was found in fasting glucose, total and low-density lipoprotein (LDL)-cholesterol (Table 2). HbA1c increased with increasing age in both genders but the trend did not reach significance in women. Higher levels of lipids, fasting glucose, HbA1c, and hsCRP were seen in women than in men, but uric acid was higher in men than in women (Table 2).

### Prevalence of general overweight/obesity and abdominal obesity among the elderly population

SHECHS participants had a high prevalence of general overweight with an overall prevalence of BMI ≥24 kg/m2 of 56.2% with no significant difference between genders. A significant reduction in the prevalence of overweight was seen with increasing age in men (p = 0.022) but not in women. The prevalence of obesity was relatively low with an overall prevalence of BMI ≥28 kg/m2 of 14.4%, and this tended to be higher in women (16.2%) than in men (12.1%). There was a non-significant tendency for a lower prevalence of obesity with increasing age in men but not in women (Table 3). A similar trend was found in the prevalence of overweight or obesity according to WHO standards with an overall prevalence of 43.1% of BMI ≥25 kg/m2 including 41.9% of men and 44.1% of women, while only 6.2% of participants had BMI ≥30 kg/m2 including 3.6% of men and 7.7% of women (data not shown).

The prevalence of abdominal obesity defined by WC ≥90 cm in men and ≥85 cm in women was 50.1% overall, being higher in women (53.8%) than in men (45.3%, P < 0.05) and a significant increase in prevalence was seen with increasing age in both genders (Table 3). The prevalence of abdominal obesity as defined by a conventional cut-off for WHtR ≥0.50^25^ was 77.1% overall, being significantly greater in women (81.2%) than in men (71.8%, P < 0.001). A significant increase in the prevalence of WHtR ≥0.50 was found with increasing age in both genders, especially in women, reaching 91.7% in the women over 80 years (Table 3, P < 0.001). This very high prevalence of WHtR ≥0.50 in the very elderly suggested this might not be an optimal cut-off point to identify increased CVD risk in this elderly population and hence the ROC analysis of WHtR and score of 10-year estimated risk of ischaemic CVD was performed. The area under the ROC curve (AUC) and its 95% CIs for the WHtR cut-off point of 0.53 were 70.3% and 68.7%–71.9%, respectively, which resulted in the highest Youden index (0.29) in the total group with a corresponding sensitivity of 69.3% and specificity of 60.0% (Supplemental Figure 1). Using the WHtR value of 0.53 as the cut-off point, the prevalence of abdominal obesity was 55.8% overall, being significantly greater in women (63.1%) than in men (46.5%, P < 0.001) and a significant increase in prevalence with increasing age was seen in both genders (Table 3, p < 0.001).

### Multivariate risk assessment

In the multivariate and multinomial logistic regression models we found that general overweight and obesity defined by BMI ≥24 kg/m2 was not significantly associated with the risk of ASCVD, but it was significantly associated with higher levels of fasting glucose, triglycerides, LDL-cholesterol, uric acid, hsCRP, increased SBP and lower education level, whereas older age was associated with reduced BMI (Table 4). Abdominal obesity defined by WC ≥90 cm in men and ≥85 cm in women was significantly associated with older age, increased risk of ASCVD and higher levels of the risk factors including fasting glucose, triglycerides, LDL-cholesterol, uric acid, hsCRP, increased SBP, and lower education level. A WHtR ≥0.53 showed a higher value for the odds ratio than high WC for the risk of ASCVD and it was the only obesity index showing significant negative association with higher physical activity level (Table 4). The analysis was repeated in subjects ≥80 years old and a similar pattern of associations was seen with WHtR ≥0.53 having the highest odds ratio (1.42) for ASCVD of all the obesity indices but this did not reach significance, probably because of the smaller sample size (supplementary table). This suggested that abdominal obesity indices might be better markers than the general obesity index of BMI for the increased risk of ASCVD and WHtR may be a better marker than WC in this elderly Chinese community population.

## Discussion

There was a high prevalence of ASCVD and overweight among the elderly SHECHS participants. The obesity indices BMI, WC and WHtR were all significantly associated with higher levels of ASCVD risk factors including fasting glucose, triglycerides, LDL-cholesterol, uric acid, hsCRP, increased SBP and lower education level, but only the abdominal obesity indices WC and WHtR were significantly associated with clinical ASCVD. A WHtR ≥0.53 had the highest odds ratio for the association with clinical ASCVD and was the only obesity index associated with the physical activity level, emphasizing that even among the very elderly individuals, abdominal obesity defined by WHtR is strongly associated with ASCVD and its risk factors. Thus, measurement of WC in addition to BMI and calculation of WHtR provides additional information about risk stratification that can be used in community public health prevention in the elderly.

BMI is a validated and widely accepted parameter to define the degree of general obesity and demonstrates significant associations with CVDs^1^,^3^,^4^,^5^,^6^,^7^. Excess bodyweight is a major public health concern, and two thirds of the adult population in the United States and at least half the populations of many other developed countries are currently overweight or obese^2^,^5^,^29^. The China National Hypertension Survey Follow-up Study (CHEFS) in 2000 found that the prevalence of BMI ≥25 kg/m2 in urban men and women aged between 45 and 79 years was 47.8% and 55.1%, respectively^29^. Our study found that the prevalence of BMI ≥25 kg/m2 in a community-dwelling Chinese population aged ≥65 years was 43.1% with no difference between men and women. The high prevalence of overweight and obesity raises the risk of morbidity and mortality from hypertension, dyslipidaemia, type 2 diabetes mellitus, ASCVD and many other diseases supporting the need for improving assessment of adiposity in community public health prevention programmes.

Adiposity is highly heterogeneous with age, sex and ethnic differences in body fat distribution^30^. Ageing in man is associated with considerable changes in body composition, including a gradual decrease of muscle mass, increase of fat mass and lower height due to compressed vertebral bodies, kyphosis and osteoporosis^31^,^32^. Loss of height in the elderly is also associated with increased morbidity and mortality^33^. BMI is widely used to assess overweight and obesity, but it does not distinguish fat from muscle or different fat distributions, thus it may not be a very accurate indicator of excess adipose tissue, especially abdominal adiposity, and associated cardiovascular risk in the elderly. This may explain why some studies found a paradoxical relationship between higher BMI and better outcome in elderly subjects. A study in Hong Kong showed that the relationship between BMI and mortality in the elderly varied according to the underlying health status^34^. Higher BMI was associated with better outcome in those with poor health status, but with worse outcome in those with initially good health status. Another study of elderly subjects (mean age 73.1 ± 66.6 years) in Taipei showed the lowest total mortality and cardiovascular mortality was in subjects who were overweight or with grade 1 obesity (BMI 25–35 kg/m2) and the risk increased in those who were underweight or severely obese^20^.

Our study found significant reductions of body weight and height with increasing age in both genders (Table 2) which probably represents loss of muscle mass and bone structure in the elderly rather than reduced adiposity. With increasing age there was a reduction in BMI in men and no significant change in women, but the prevalence of ASCVD increased and BMI was not significantly associated with the presence of ASCVD (Table 4), suggesting BMI is a less than optimal risk assessment tool for predicting ASCVD in the elderly.

Abdominal obesity defined by WC or WHtR more effectively reflects the visceral fat and has been shown to be more strongly associated than BMI with CVDs and overall mortality^8^,^9^,^10^,^11^,^12^. Most studies used general populations which generally did not include the very elderly. A study in Hong Kong^8^ included subjects between 25 and 74 years, a German study used a general population aged 18 and over with no age strata after 66 years old^9^, a study in Bangladesh^10^ included 562 middle-aged participants and the Beijing study examined the general population aged 18–79 years^12^. One elderly cohort was the Mediterranean study which analyzed men aged 55–80 years and women aged 60–80 years but only those at high cardiovascular risk and initially free of CVD^11^. Our study enrolled participants from an urban community to observe the elderly population and found WC increased significantly with age only in women whereas WHtR increased significantly with age in both genders although there was reduction of body weight and height with increasing age and a significant decline in BMI only in men (Table 2). The reduction in body height with age might tend to exaggerate the increase in obesity measured by BMI or WHtR, but WHtR retained the highest odds ratio for ASCVD even in subjects aged over 80 years and it was the only obesity index significantly associated with physical activity.

There was a very high prevalence of abdominal obesity defined by WHtR ≥0.50 at 91.7% in women and 74.9% in men among those very elderly subjects, suggesting the WHtR cut-off point 0.50 might not be optimal in this elderly population. The cut-off point of 0.50 or lower has been identified in other large studies to predict the risk of CVD risk factors in Chinese but lower values may be more appropriate for younger age groups^15^. We performed the ROC analysis of WHtR and the validated score of 10-year estimated risk of ischaemic CVD^28^ and found that a WHtR ≥0.53 had the highest Youden index (0.29) with a corresponding sensitivity of 69.3% and specificity of 60.0%. This study examined an elderly population including 608 individuals aged over 80 years and demonstrated that the abdominal obesity indices WC and WHtR were better associated with ASCVD than BMI.

Lifestyle intervention with exercise was reported to reduce abdominal obesity and improve coronary heart disease risk factors^35^. Our study indicated that WHtR was the only obesity index showing a significant association with physical activity suggesting that it might be a more sensitive index for assessment of ASCVD risk and the response to exercise intervention in this elderly population.

Both general overweight or obesity and abdominal obesity in this study were found to be significantly associated with low level of education in the elderly population, therefore future education for better lifestyle should be recommended to reduce the prevalence of overweight and obesity to decrease the CVD burden.

This study has several limitations including that it currently provides only cross-sectional data which cannot prove that abdominal obesity is a causative risk factor for CVD. Indeed, there could be reverse causality in the associations demonstrated between obesity and ASCVD, but this seems unlikely as obesity was also associated with the risk factors for ASCVD and other prospective studies have shown that obesity predicts the development of ASCVD^13^ . The prevalence of CVD and the CVD risk factors in the population might be underestimated in this study because the individuals were invited to attend the health examination and this may result in bias with healthier subjects joining the examination. Our future follow-up study and studies in other regions will provide more information about the association of general or abdominal obesity and increased ASCVD events and risk and mortality, and also whether better lifestyle can reduce the prevalence of overweight and obesity with educational advice in these elderly individuals.

In summary, our results showed that general overweight and abdominal obesity were highly prevalent in the SHECHS participants and abdominal obesity and in particular a WHtR value above 0.53 was strongly associated with prevalent ASCVD and its risk factors with a higher value for odds ratio than WC and BMI in these elderly Chinese subjects in the community.

## Additional Information

**How to cite this article**: Fan, H. *et al*. Abdominal obesity is strongly associated with Cardiovascular Disease and its Risk Factors in Elderly and very Elderly Community-dwelling Chinese. *Sci. Rep.* **6**, 21521; doi: 10.1038/srep21521 (2016).

## Supplementary Material

Supplementary Information

## Figures and Tables

**Table 1:** Demographic and clinical characteristics of elderly Chinese subjects stratified by gender.

	**Men**	**Women**	**Total**	**P value**
	**n** = **1745**	**n** = **2205**	**n** = **3950**	
Gender (%)	44.22	55.81		
Age (years)	71.76 (71.46–72.07)	72.11 (71.83–72.39)	71.96 (71.75–72.16)	0.097
Education level, % (n)				<0.001
<6 years	24.34 (425)	55.51 (1224)	41.74 (1649)	
6–12years	58.48 (1021)	39.86 (879)	48.09 (1900)	
>12 years	17.18 (300)	4.63 (102)	10.17 (402)	
Health habits				
Current cigarette user, % (n)	30.58 (534)	1.00 (22)	14.07 (556)	<0.001
Current alcohol user, % (n)	29.32 (512)	1.36 (30)	13.71 (542)	<0.001
Physical activity, % (n)				<0.001
Active	28.46 (497)	21.90 (483)	24.80 (980)	
Not active	71.56 (1249)	79.10 (1722)	75.20 (2971)	
Hypertension, % (n)	73.94 (1291)	75.46 (1664)	74.79 (2955)	0.273
Diabetes, % (n)	19.70 (344)	21.54 (475)	20.73 (819)	0.156
Clinical ASCVD^†^, % (n)	22.10 (386)	21.04 (464)	21.51 (850)	0.419
SBP, mmHg	138.4 (137.6–139.3)	139.1 (138.4–139.8)	138.8 (138.3–139.3)	0.237
DBP, mmHg	82.54 (82.10–82.97))	81.34 (80.98–81.70)	81.87 (81.59–82.14)	<0.001
Body weight, kg	68.36 (67.90–68.82)	59.57 (59.17–59.96)	63.45 (63.12–63.78)	<0.001
Height, cm	167.1 (166.8–167.4)	155.1 (154.8–155.3)	160.4 (160.1–160.6)	<0.001
BMI, kg/m2	24.46(24.31–24.61)	24.74(24.59–24.89)	24.62(24.51–24.72)	0.011
Waist circumference, cm	88.11(87.67–88.55)	85.58 (85.19–85.97)	86.70 (86.41–86.99)	<0.001
WHtR	0.52 (0.52–0.56)	0.55 (0.54–0.55)	0.54 (0.53–0.54)	<0.001
FG, mmol/L	5.68 (5.59–5.76)	5.82 (5.74–5.90)	5.76 (5.70–5.81)	0.018
HbA1c, %	6.30 (6.25–6.35)	6.39 (6.35–6.44)	6.35 (6.32–6.39)	0.006
TG, mmol/L	1.53 (1.47–1.58)	1.71 (1.66–1.76)	1.63 (1.59–1.66)	<0.001
TC, mmol/L	4.72 (4.67–4.76)	5.20 (5.16–5.24)	4.98 (4.95–5.01)	<0.001
LDL–C, mmol/L	3.12 (3.08–3.16)	3.45 (3.41–3.49)	3.30 (3.28–3.33)	<0.001
HDL–C, mmol/L	1.38 (1.36–1.40 )	1.52 (1.51–1.54)	1.46 (1.45–1.47)	<0.001
Uric acid, μmol/L	357.0 (353.0–361.1)	305.4 (302.0–308.8)	328.2 (325.5–330.9)	<0.001
hsCRP, mg/L	1.48 (1.40–1.56)	1.66 (1.59–1.74)	1.58 (1.53–1.63)	0.001
Creatinine, μmol/L	87.23 (85.96–88.50)	68.60 (67.77–69.44)	76.83 (76.05–77.62)	<0.001

Values are mean (95% confidence intervals) or percentages % (number). P values comparing men and women.

Clinical atherosclerotic cardiovascular disease (ASCVD) includes established CVD plus stable or unstable angina and transient ischemic attack; SBP, systolic blood pressure; DBP, diastolic blood pressure; BMI, body mass index; WC, waist circumference; WHtR, waist–to-height ratio; TC, total cholesterol; LDL-C, low–density lipoprotein cholesterol; HDL–C, high–density lipoprotein cholesterol; TG, triglycerides; FG, fasting glucose; hsCRP, high sensitivity C reactive protein.

**Table 2:** Obesity indices and metabolic variables in elderly Chinese subjects stratified by age and gender.

**Age (years)**	**65**–**69**	**70**–**79**	**≥80**	**Total**	**P value**
Men, n	831	667	247	1745	
ASCVD, % (n)	17.56 (146)	24.46 (163)	31.17 (77)	22.10 (386)	<0.001
Current cigarette user, % (n)	36.5 (303)	27.6 (184)	19.0 (47)	30.6 (534)	<0.001
Current alcohol user, % (n)	31.8 (264)	28.3 (189)	23.9 (59)	29.3 (512)	0.013
Body weight, (kg)	69.87 (69.21–70.53)	67.92 (67.19–68.64)	64.49 (63.27–65.71)	68.36 (67.90–68.82)	<0.001
Height, cm	168.4 (167.9–168.8)	166.6 (166.1–167.1)	164.0 (163.1–164.8)	167.1 (166.8–167.4)	<0.001
BMI, kg/m2	24.62 (24.41–24.83)	24.45 (24.21–24.69)	23.95 (23.55–24.35)	24.46(24.31–24.61)	0.013
WC, cm	87.64 (86.99–88.30)	88.43 (87.73–89.13)	88.83 (87.65–90.01)	88.11(87.67–88.55)	0.12
WHtR	0.52 (0.51–0.52)	0.53 (0.52–0.53)	0.54 (0.53–0.54)	0.52 (0.52–0.53)	<0.001
SBP, mmHg	135.9 (134.7–137.07)	140.1 (138.8–141.4)	142.5 (140.2–144.7)	138.4 (137.6–139.3)	<0.001
DBP, mmHg	83.38 (82.76–84.01)	81.98 (81.30–82.67)	81.17 (79.98–82.36)	82.54 (82.10–82.97)	0.001
FG, mmol/L	5.65 (5.53–5.77)	5.67 (5.54–5.80)	5.78 (5.48–6.07)	5.68 (5.59–5.76)	0.634
HbA1c, %	6.27 (6.20–6.34)	6.27 (6.19–6.35)	6.47 (6.29–6.65)	6.30 (6.25–6.35)	0.028
TG, mmol/L	1.64 (1.56–1.73)	1.45 (1.38–1.51)	1.33 (1.23–1.43)	1.53 (1.47–1.58)	<0.001
TC, mmol/L	4.73 (4.67–4.79)	4.71 (4.63–4.78)	4.70 (4.58–4.82)	4.72 (4.67–4.76)	0.838
LDL–C, mmol/L	3.12 (3.06–3.18)	3.12 (3.05–3.18)	3.09 (2.99–3.20)	3.12 (3.08–3.16)	0.880
HDL–C, mmol/L	1.35 (1.32–1.37)	1.39 (1.36–1.42)	1.47 (1.42–1.52)	1.38 (1.36–1.40 )	<0.001
Uric acid, μmol/L	354.1 (348.4–359.9)	354.1 (348.4–361.1)	372.9.1 (361.5–384.3)	357.0 (353.0–361.1)	0.007
hsCRP, mg/L	1.38 (1.27–1.49)	1.59 (1.46–1.72)	1.49 (1.29–1.69)	1.48 (1.40–1.56)	0.041
Women, n	1019	825	361	2205	
ASCVD, % (n)	16.29 (166)	25.45 (210)	24.37 (88)	21.04 (464)	<0.001
Current cigarette user, % (n)	0.5 (5)	1.2 (10)	1.9 (7)	1.0 (22)	0.066
Current alcohol user, % (n)	1.1 (11)	1.6 (13)	1.7 (6)	1.4 (30)	0.321
Body weight, (kg)	60.90 (60.33–61.47)	59.33 (58.70–59.95)	56.33 (55.25–57.41)	59.57 (59.17–59.96)	<0.001
Height, cm	156.8 (156.5–157.2)	154.5 (154.1–154.9)	151.3 (150.7–152.0)	155.1 (154.8–155.3)	<0.001
BMI, kg/m2	24.73 (24.52–24.94)	24.83 (24.58–25.08)	24.54 (24.13–24.96)	24.74(24.59–24.89)	0.429
WC, cm	84.32 (83.78–88.30)‡	86.09 (85.45–86.73)†	87.96 (86.94–89.00 )	85.58 (85.19–85.97)‡	<0.001
WHtR	0.53 (0.53–0.54)‡	0.55 (0.55–0.56)‡	0.58 (0.57–0.58)‡	0.55 (0.54–0.55)‡	<0.001
SBP, mmHg	136.9 (135.9–137.9)	140.8 (139.6–142.1)	141.4 (139.7–143.0)	139.1 (138.4–139.8)	<0.001
DBP, mmHg	81.76 (81.24–82.29)†	81.08 (80.48–81.67)	80.75 (79.83–81.66)	81.34 (80.98–81.70)†	0.086
FG, mmol/L	5.75 (5.64–5.86)†	5.86 (5.72–5.99)†	5.93 (5.73–6.14)†	5.82 (5.74–5.90)†	0.220
HbA1c, %	6.36 (6.29–6.42)†	6.43 (6.35–6.51)†	6.42 (6.31–6.54)	6.39 (6.35–6.44)†	0.138
TG, mmol/L	1.75 (1.66–1.84)†	1.73 (1.65–1.80)‡	1.55 (1.47–1.63)†	1.71 (1.66–1.76)‡	0.025
TC, mmol/L	5.22 (5.17–5.28)‡	5.19 (5.12–5.25)‡	5.14 (5.03–5.24)‡	5.20 (5.16–5.24)‡	0.310
LDL–C, mmol/L	3.46 (3.41–3.51)‡	3.46 (3.40–3.53)‡	3.40 (3.31–3.49)‡	3.45 (3.41–3.49)‡	0.455
HDL–C, mmol/L	1.52(1.50–1.55)‡	1.51(1.48–1.53)‡	1.57(1.52–1.61)†	1.52 (1.51–1.54)‡	0.047
Uric acid μmol/L	293.3 (288.8–297.8)‡	310.4 (304.9–315.9)‡	328.0 (318.1–337.9)‡	305.4 (302.0–308.8)‡	<0.001
hsCRP, mg/L	1.55 (1.44–1.65)†	1.73 (1.61–1.85)	1.82 (1.62–2.03)†	1.66 (1.59-1.74)†	0.015

Values are mean (95% confidence intervals) or percentages % (number). P values comparing age groups by ANOVA.

Clinical atherosclerotic cardiovascular disease (ASCVD) includes established CVD plus stable or unstable angina and transient ischemic attack; BMI, body mass index; WC, waist circumference; WHtR, waist-to-height ratio; SBP, systolic blood pressure; DBP, diastolic blood pressure; FG, fast glucose; TG, triglycerides; TC, total cholesterol; LDL-C, low density lipoprotein; HDL-C, high density lipoprotein cholesterol; ALT, alanine transaminase; hsCRP, high sensitivity C reactive protein.

^†^Statistically significantly different from men, p < 0.05.

^‡^Statistically significantly different from men, p < 0.001.

**Table 3:** Age- and sex-standardized prevalence of general overweight/obesity and abdominal obesity.

	**65**–**69 yr**	**70**–**79 yr**	**≥ 80 yr**	**Total**	**P value**
Men, n	831	667	247	1745	
BMI, kg/m2					
≧24.0	58.1 (483)	55.5 (370)	48.2 (119)	55.7 (972)	0.022
≧28.0	12.4 (103)	13.0 (87)	8.9 (22)	12.1 (212)	0.225
WC, cm					
≧90 cm	42.1 (350)	48.6 (323)	47.4 (117)	45.3 (790)	0.035
WHtR					
≧0.50	69.0 (573)	74.1 (492)	74.9 (185)	71.8 (1250)	0.045
≧0.53	40.6 (337)	50.0 (332)	57.1 (141)	46.5 (810)	<0.001
Women, n	1019	825	361	2205	
BMI, kg/m2					
≧24.0	56.3 (574)	57.1 (471)	56.0 (202)	56.6 (1247)	0.918
≧28.0	16.3 (166)	15.9 (131)	16.6 (60)	16.2 (357)	0.944
WC, cm					
≧85 cm	48.7 (496)†	56.3 (464)‡	62.5 (225)‡	53.8 (1185)‡	0.001
WHtR					
≧0.50	76.2 (776)‡	82.9 (683)‡	91.7 (330)‡	81.2 (1789)‡	<0.001
≧0.53	55.3 (563)‡	66.9 (547)‡	77.5 (279)‡	63.1 (1389)‡	<0.001
Overall, n	1850	1492	608	3950	
BMI, kg/m2					
≧24.0	57.1 (1057)	56.4 (841)	52.8 (321)	56.2 (2219)	0.171
≧28.0	14.5 (269)	14.6 (218)	13.5 (82)	14.4 (569)	0.781
WC, cm					
≧90M/ 85W	45.8 (846)	52.9 (787)	56.3 (342)	50.1 (1975)	<0.001
WHtR					
≧0.50	73.0 (1349)	79.0 (1175)	84.8 (515)	77.1 (3039)	<0.001
≧0.53	48.7 (900)	59.1 (879)	69.2 (420)	55.8 (2199)	<0.001

BMI, body mass index; WC, waist circumference; WHtR, waist–to–height ratio.

^†^Statistically significantly different from men, p < 0.05.

^‡^Significantly different from men, p < 0.001.

**Table 4:** Multivariable–adjusted odds ratios of WHtR, WC and BMI models.

**Variable**	**BMI ≥24**		**WC ≥90 Men /85 Women**		**WHtR ≥0.530**	
	**Odds ratio**	**p value**	**Odds ratio**	**p value**	**Odds ratio**	**p value**
Age	0.97 (0.96–0.98)	<0.001	1.00 (1.00–1.02)	0.031	1.02 (1.00–1.03)	0.002
Male sex	0.89 (0.73–1.07)	0.230	1.27 (1.05–1.54)	0.012	1.69 (1.39–2.05)	<0.001
**ASCVD**	**1.11 (0.94**–**1.33)**	**0.221**	**1.20 (1.01**–**1.43)**	**0.036**	**1.31 (1.09**–**1.57)**	**0.003**
FG, mmol/L	1.16 (1.08–1.24)	<0.001	1.17 (1.09–1.25)	<0.001	1.13 (1.06–1.22)	<0.001
HbA1c, %	1.25 (1.09–1.43)	0.001	1.19 (1.04–1.36)	0.008	1.13 (1.00–1.30)	0.042
TG, mmol/L	1.20 (1.11–1.31)	<0.001	1.17 (1.08–1.26)	<0.001	1.21 (1.12–1.32)	<0.001
LDL–C, mmol/L	1.08 (1.00–1.18)	0.046	1.10 (1.01–1.20)	0.018	1.08 (1.00–1.17)	0.046
Uric Acid, μmol/L	1.00 (1.00–1.01)	<0.001	1.00 (1.00–1.01)	<0.001	1.00 (1.00–1.01)	<0.001
hsCRP, mg/L	1.12 (1.07–1.17)	0.017	1.12 (1.07–1.18)	0.002	1.13 (1.08–1.18)	<0.001
SBP, mmHg	1.01 (1.01–1.02)	<0.001	1.00 (1.00–1.01)	<0.001	1.00 (1.00–1.01)	<0.001
Education	0.81 (0.74–0.88)	<0.001	0.84 (0.77–0.91)	<0.001	0.75 (0.69–0.82)	<0.001
Current cigarette user	0.64 (0.51–0.81)	<0.001	0.72 (0.57–0.91)	0.006	0.77 (0.61–0.98)	0.032
Current alcohol user	1.02 (0.82–1.29)	0.804	1.06 (0.84–1.33)	0.614	0.97 (0.77–1.22)	0.817
Physical activity	**1.07 (0.93**–**1.23)**	**0.290**	**0.92 (0.79**–**1.05)**	**0.210**	**0.85 (0.73**–**0.97)**	**0.023**

Adjusted variables included Age, Sex, ASCVD, FG, HbA1c, TG, LDL–C, Uric Acid, SBP, Education, Current cigarette user, Current alcohol user and Physical activity.

ASCVD, atherosclerotic cardiovascular disease; BMI, body mass index; WC, waist circumference; WHtR, waist–to-height ratio; SBP, systolic blood pressure; FG, fast glucose; TG, triglycerides; LDL–C, low density lipoprotein; hsCRP, high sensitivity C reactive protein.
