# Supplementary material for: Abdominal obesity is strongly associated with Cardiovascular Disease and its Risk Factors in Elderly and very Elderly Community-dwelling Chinese
Source: Sci Rep. 2016 Feb 17;6:21521. doi: 10.1038/srep21521 (PMC4756331; doi:10.1038/srep21521)
Supplement: Supplementary Information [file srep21521-s1.doc]

**Abdominal obesity is strongly associated with Cardiovascular Disease and its Risk Factors in Elderly and very Elderly Community-dwelling Chinese**

Huimin Fan*^1^, Xiaolin Li*^1^, Liang Zheng*^1^, Xiaoli Chen^1^, Qin lan^1^, Hong Wu^2^, Xugang Ding^2^, Dingguang Qian^2^, Yixin Shen^1^, ZuorenYu^1^, Lieying Fan^1^, Ming Chen^1^, Brian Tomlinson^3^, Paul Chan^4^, Yuzhen Zhang^§1^, Zhongmin Liu^§1^

1, Research Center for Translational Medicine, Key Laboratory of Arrhythmias, Ministry of Education, Shanghai East Hospital, Tongji University School of Medicine, Shanghai 200120, China

2, Gaohang Community Medical Center, 180 Gingao Road, Pudong New area, Shanghai, 200120, China

3, Department of Medicine and Therapeutics, The Chinese University of Hong Kong, Hong Kong SAR, China

4, Division of Cardiology, Department of Internal Medicine, Wan Fang Hospital, Taipei Medical University, Taipei, Taiwan

**Supplement table: Multivariable-adjusted odds ratios of WHtR, WC and BMI models**

**in subjects over 80 years old**

| Variable | BMI ≥24 | | WC ≥90 Men /85 Women | | WHtR ≥0.530 | |
| --- | --- | --- | --- | --- | --- | --- |
|  | Odds ratio | p value | Odds ratio | p value | Odds ratio | p value |
| Age | 0.97 (0.91-1.04) | 0.463 | 1.00 (0.94-1.07) | 0.915 | 1.02 (0.97-2.90) | 0.480 |
| Male sex | 1.00 (0.58-1.73) | 0.992 | 1.13 (0.66-1.94) | 0.644 | 1.68 (0.97-2.90) | 0.060 |
| ASCVD | 1.46 (0.94-2.26) | 0.086 | 1.35 (0.87-2.08) | 0.175 | 1.38 (0.87-2.19) | 0.167 |
| FG, mmol/L | 1.13 (0.94-1.37) | 0.168 | 1.21 (1.00-1.47) | 0.049 | 1.03 (0.95-1.26) | 0.073 |
| HbA1c, % | 1.42 (0.97-2.08) | 0.065 | 1.28 (0.98-1.87) | 0.064 | 1.32 (0.98-1.96) | 0.074 |
| TG, mmol/L | 1.53 (1.12-2.09) | 0.007 | 1.42 (0.99-1.61) | 0.056 | 1.42 (1.02-1.97) | 0.034 |
| LDL-C, mmol/L | 1.09 (0.86-1.38) | 0.471 | 1.21 (0.96-1.53) | 0.109 | 0.98 (0.76-1.25) | 0.848 |
| Uric Acid, µmol/L | 1.00 (1.00-1.01) | <0.001 | 1.00 (1.00-1.01) | 0.003 | 1.00 (0.99-1.00) | 0.127 |
| hsCRP, mg/L | 1.14 (0.98-1.29) | 0.130 | 1.10 (0.98-1.25) | 0.089 | 1.07 (0.97-1.21) | 0.194 |
| SBP, mmHg | 1.00 (0.99-1.01) | 0.185 | 1.00 (0.99-1.01) | 0.190 | 1.00 (0.99-1.01) | 0.196 |
| Education | 0.82 (0.64-1.05) | 0.131 | 0.74 (0.57-0.94) | 0.015 | 0.72 (0.56-0.91) | 0.008 |
| Current cigarette user | 0.72 (0.35-1.47) | 0.374 | 0.77 (0.38-1.56) | 0.475 | 0.76 (0.38-1.51) | 0.437 |
| Current alcohol user | 0.89 (0.47-1.69) | 0.736 | 0.71 (0.37-1.33) | 0.290 | 0.83 (0.44-1.54) | 0.561 |
| Physical activity | 0.87 (0.59-1.28) | 0.501 | 1.00 (0.68-1.46) | 0.985 | 0.86 (0.58-1.28) | 0.475 |

Adjusted variables included Age, Sex, ASCVD, FG, HbA1c, TG, LDL-C, Uric Acid, SBP, Education and Physical activity.

ASCVD, atherosclerotic cardiovascular disease; BMI, body mass index; WC, waist circumference; WHtR, waist-to-height ratio; SBP, systolic blood pressure; FG, fast glucose; TG, triglycerides; LDL-C, low density lipoprotein; hsCRP, high sensitivity C reactive protein.

**
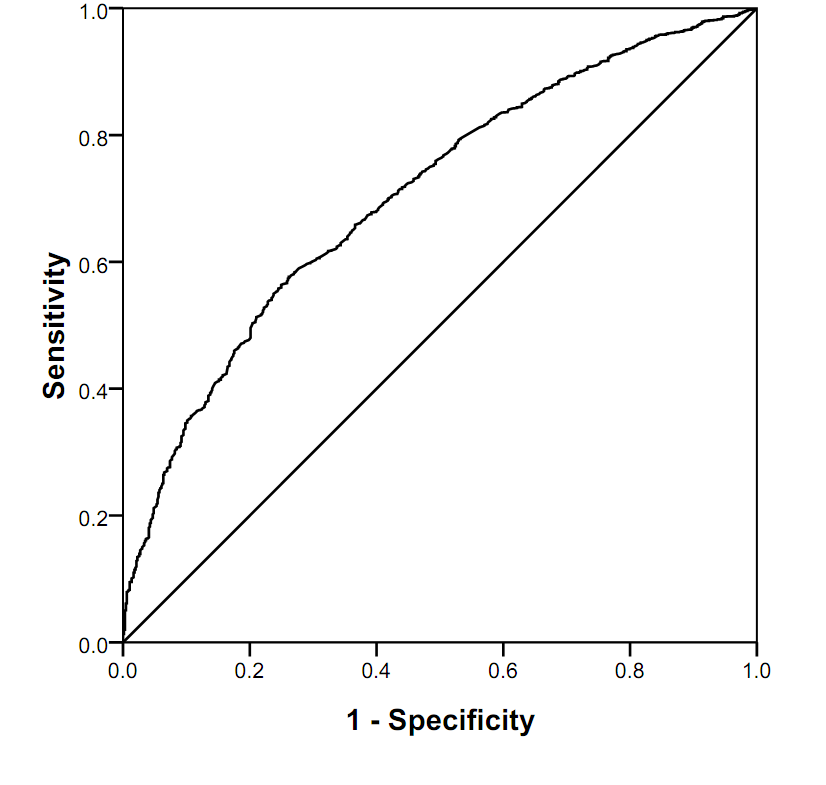
**

**Supplement Figure 1: ROC analysis of waist-to-height ratio and score of 10-year estimated risk of ischemic cardiovascular diseases.** AUC = 70.3%; 95% CI 68.7% - 71.9%; cut-off point = 0.53; sensitivity = 69.3%, specificity =60.0% and Youden index = 0.29.
